# Manual and ventilator hyperinflation parameters used by intensive care physiotherapists in Sri Lanka: An online survey

**DOI:** 10.1371/journal.pone.0297880

**Published:** 2024-05-20

**Authors:** Indrajith Liyanage, D. A. R. K. Dassanayaka, F. M. D. Chellapillai, E. Liyanage, S. Rathnayake, M. Rikas, S. Mayooran

**Affiliations:** 1 Department of Physiotherapy, Faculty of Allied Health Sciences, University of Peradeniya, Peradeniya, Sri Lanka; 2 Department of Physiotherapy, Faculty of Allied Health Sciences, General Sir John Kotelawala Defence University, Dehiwala-Mount Lavinia, Sri Lanka; 3 Department of Nursing, Faculty of Allied Health Sciences, University of Peradeniya, Peradeniya, Sri Lanka; University of Sharjah, UNITED ARAB EMIRATES

## Abstract

**Introduction:**

Hyperinflation is a common procedure to clear secretion, increase lung compliance and enhance oxygenation in mechanically ventilated patients. Hyperinflation can be provided as manual hyperinflation (MHI) or ventilator hyperinflation (VHI), where outcomes depend upon the methods of application. Hence it is crucial to assess the application of techniques employed in Sri Lanka due to observed variations from recommended practices.

**Objective:**

This study is aimed to evaluate the application and parameters used for MHI and VHI by physiotherapists in intensive care units (ICUs) in Sri Lanka.

**Methodology:**

An online survey was conducted among physiotherapists who are working in ICUs in Sri Lanka using WhatsApp groups and other social media platforms.

**Results:**

A total of 96 physiotherapists responded. The survey comprised of three sections to obtain information about socio-demographic data, MHI practices and VHI practices. Most of the respondents (47%) worked in general hospitals and 74% of participants had a bachelor’s degree in physiotherapy; 31.3% had 3–6 years of experience; 93.8% used hyperinflation, and 78.9% used MHI. MHI was performed routinely and as needed to treat low oxygen levels, abnormal breath sounds, and per physician orders while avoiding contraindications. Self-inflation bags are frequently used for MHI (40.6%). Only a few participants (26%) used a manometer or tracked PIP. In addition to the supine position, some participants (37.5%) used the side-lying position. Most physiotherapists followed the recommended MHI technique: slow squeeze (57.3%), inspiratory pause (45.8%), and quick release (70.8%). VHI was practised by 19.8%, with medical approval and it was frequently performed by medical staff compared to physiotherapists. Treatment time, number of breaths, and patient positioning varied, and parameters were not well-defined.

**Conclusion:**

The study found that MHI was not applied with the recommended PIP, and VHI parameters were not identified. The study indicates a need to educate physiotherapists about current VHI and MHI practice guidelines.

## Introduction and literature review

Mechanical ventilation is the most widely used short-term life support technique for a wide range of medical and surgical conditions [1, 2]. The use of mechanical ventilation in critically ill patients is associated with the development of pulmonary complications. Hyperinflation is a routinely used method to clear secretion in the lung, improve oxygenation, and improve lung compliance in mechanically ventilated patients [3–9]. The principle of hyperinflation is to deliver a breath larger than the tidal volume of the patient [10] with an inspiratory pause and increased expiratory flow [6, 11, 12].

Hyperinflation can be applied in two ways either manually (MHI) or mechanically using a ventilator (VHI). During MHI, secretion clearance of the lung occurs as a result of increased expiratory flow, which is explained by the two-phase gas-liquid exchange theory [13]. The two-phase gas-liquid exchange theory states that the peak expiratory flow rate (PEFR) should be 10% higher than the peak inspiratory flow rate (PIFR), and at least PEFR should be 1000 cm/second to clear the secretion [14]. Other than the expiratory flow rate, it is imperative not to exceed the peak inspiratory pressure (PIP) of more than 40 cmH_2_O to prevent lung injuries [15]. Therefore, it is also advised to use a manometer in the ventilatory circuit to measure the PIP [16]. The studies conducted in the United Kingdom have reported that 32% of the physiothearpists used MHI without a manometer and 41% with a manometer [17]. An Australian study reported that 31% of physiotherapists used a manometer and 51% set PIP < 40cmH_2_O [18]. This study further reported that a high PEFR is obtained by the slow squeeze during inspiration and quick release during expiration in manual hyperinflation. In Australia, majority of the physiotherapists (96%) employed the MHI technique, implementing it through a slow inspiration, inspiratory hold, followed by rapid expiration [16]. However, in the United Kingdom, a lower adherence rate of less than 50% to the recommended guidelines was identified for this technique, as reported by [19]. It is suggested that during MHI most clinical settings use higher PIFR than PEFR [20, 21].

VHI is a relatively newer technique and uses different ventilator settings to give breaths larger than the normal tidal volume. This method does not require the patient to be disconnected from the ventilator and has an advantage over MHI in providing hyperinflation [22] for high positive-end expiratory pressure (PEEP) dependent patients. The VHI can be performed using different ventilator modes [23], such as, synchronised intermittent mandatory ventilation (SIMV) volume control mode (VC) or pressure control mode (PC) [22]. In VHI, the ventilator is set to deliver hyperinflation followed by suctioning. This is a safe and effective way of secretion removal [24]. Currently, there are no established recommendations for optimal ventilator settings when conducting the technique [25]. However, there is a large variation in the way the technique is applied [9, 21] and in terms of ventilator mode. Nevertheless, literature suggests that SIMV-VC is more successful than PSV and PC-SIMV modes [26].

Even though the MHI technique is the most commonly used (84%) hyperinflation technique in the Sri Lankan context [27], MHI parameters and their uses as well as VHI and its parameters have not been identified. The outcome of MHI and VHI highly depends upon the parameters of PIP, PEFR, PIFR, PEEP and FiO_2_ and the method of application.

### Objective of the study

This study is aimed to evaluate the methods, indications and parameters of MHI and VHI techniques used by intensive care physiotherapists in Sri Lanka.

## Methods

### Study design and setting

An online survey was conducted via a Google form. The results are presented in accordance with the Checklist for Reporting Results of Internet E-Surveys (CHERRIES) [28]. This study was conducted in public and private sector hospitals in Sri Lanka. The types of hospitals included were National Hospitals, General Hospitals, District General Hospitals, Teaching Hospitals, Provincial Hospitals, and Base Hospitals.

### Participants and recruitment

Physiotherapists working in (ICUs) in hospitals participated in this study. About 645 physiotherapists are currently practising in Sri Lanka, with 493 of them working in hospitals that have ICUs. The sample size for a study was calculated using the Solving formula, with a 10% error and a 5% significant level. After accounting for a 15% dropout rate, a total of 96 samples were required. Physiotherapists who had work experience of more than six months in ICUs were included. Participants were invited through social media (WhatsApp and Messenger).

Additionally, chief physiotherapists of the relevant hospitals were personally contacted and were requested to promote the survey. During the data collection period, three reminders were sent via social media (WhatsApp and Messenger). Repeated participation in this open online survey was prevented by automatically blocking previously used IP addresses.

### Data collection instrument

The data collection instrument was a questionnaire comprising three sections. The first section encompassed socio-demographic information, consisting of the nature of the hospital employed, years of experience working as a physiotherapist in an ICU, and the highest education qualification obtained in the field of physiotherapy.

Section two consisted of questions related to the use of MHI and was assessed by a questionnaire developed by the research team based on the literature [16, 19, 29]. This section assessed the person responsible for performing the hyperinflation; the indication or selection criteria to use hyperinflation; the aim/s of the hyperinflation technique; the device used for MHI; FiO02 used; number of breaths per set used during the application of MHI; total duration of the treatment; the position of the patient, precautions and contraindications for MHI; the technique used; using peak expiratory valve for PEEP dependent patient; setting maximum inspiratory pressure /peak inspiratory pressure during the application; and measuring PIP during MHI.

In the third section, we used a previously designed questionnaire that assessed VHI with prior permission [22] consisting of the need for medical approval to perform VHI; the staff category performing VHI; availability of VHI protocol; frequency of performing VHI; indications for performing VHI; reasons for choosing to perform VHI instead of manual hyperinflation; the modes of ventilation commonly using in performing VHI; commonly performed VHI; altering the technique of VHI when treating lung collapse versus sputum retention; maximum pressure or maximum volume not to exceed during VHI; deep breaths providing in one set; sets of deep breathe provided; and how to learn the practical aspects of performing the technique of VHI.

The MHI part of the questionnaire was created based on available literature [16, 19, 29], while the VHI part was taken from a previously used questionnaire. Both parts were reviewed by a research team comprising five academicians who are registered physiotherapists and one academician who is a registered nurse. The data collection instrument was pre-tested among a conveniently selected sample of six physiotherapists for the readability and understandability of the questions. Based on the results of the pre-test, no modifications were made.

### Data collection

Data collection was carried out for a period of three months from 28^th^ May 2022 to 30^th^ July 2022. The questionnaire along with the information sheet was shared through emails and social media.

### Data analysis

Data were first extracted into an Excel sheet and then to SPSS 25 version software. Descriptive statistics were used to characterise the sample and each item of sections two and three of the questionnaire.

### Ethical considerations

Ethical clearance was obtained from the Ethical Review Committee, Faculty of Allied Health Sciences, University of Peradeniya, Sri Lanka (AHS/ERC/2022/020). The information sheet was distributed with the online survey invitation. Only participants who consented to participate in this study filled out the questionnaire, and their consent was recorded as a part of the online survey. All data collection forms were given a code number, and coded information was used in data management.

## Results

### Socio-demographic data

In this survey, 96 physiotherapists who worked in ICUs responded. Table 1 presents the socio-demographic profile of the respondents. The majority were from General Hospitals (47%), followed by Teaching Hospitals (17%) and National Hospitals (12%) of Sri Lanka. The majority (74%) had a Bachelor’s Degree in Physiotherapy as the highest qualification. The majorityof participants (31.3%) had three to six years of experience as physiotherapists, and 26% of the sample had three to six years of experience in the ICU (Table 1).

**Table 1:** Descriptive statistics for the sociodemographic characteristics of the respondents (n = 96).

Sociodemographic of participants	Frequency or number (%)
**Type of hospital working**	
National Hospital	12 (12.5)
General Hospital	47 (49)
Provincial General Hospital	6 (6.3)
Base Hospital	7 (7.3)
Teaching Hospital	17 (17.7)
Private Hospital	6 (6.3)
Other	1 (1)
**Working Experience**	
<6 months	1 (1)
6 months—1 year	0 (0)
1–3 years	16 (16.7)
3–6 years	30 (31.3)
6–9 years	23 (24)
9–12 years	6 (6.3)
>12 years	20 (20.8)
**Working experience at ICU**	
<6 months	3 (3.1)
6 months—1 year	9 (9.4)
1–3 years	20 (20.8)
3–6 years	26 (27.1)
6–9 years	17 (17.7)
9–12 years	6 (6.3)
>12 years	15 (15.6)
**Education qualifications**	
Diploma in Physiotherapy	22 (22.9)
Bachelor’s degree in Physiotherapy	71 (74)
Master’s degree in Physiotherapy	3 (3.1)
Doctoral degree in Physiotherapy	0 (0)

### Practice of MHI

**Fig 1:**
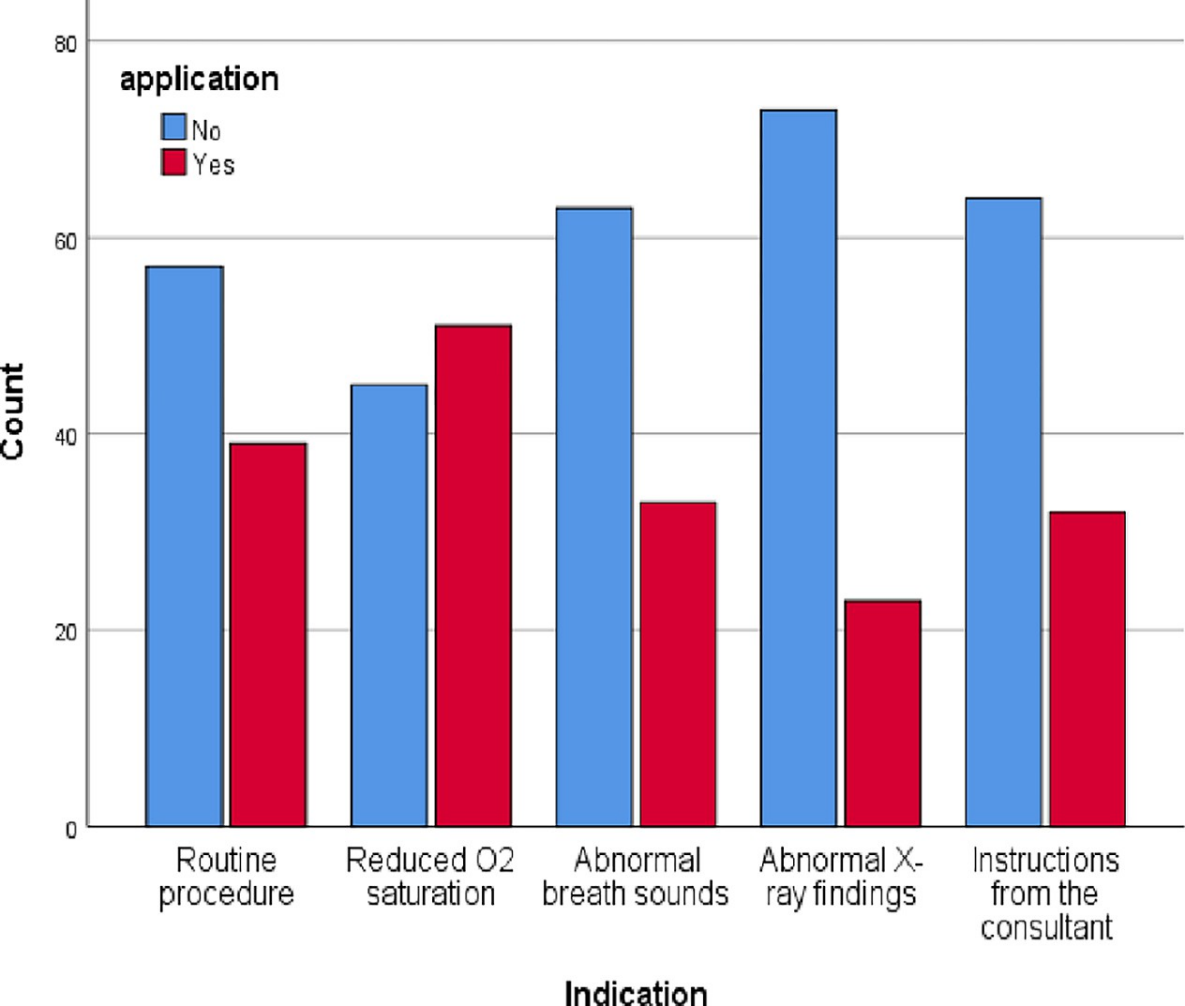
Selection criteria of MHI.

**Fig 2:**
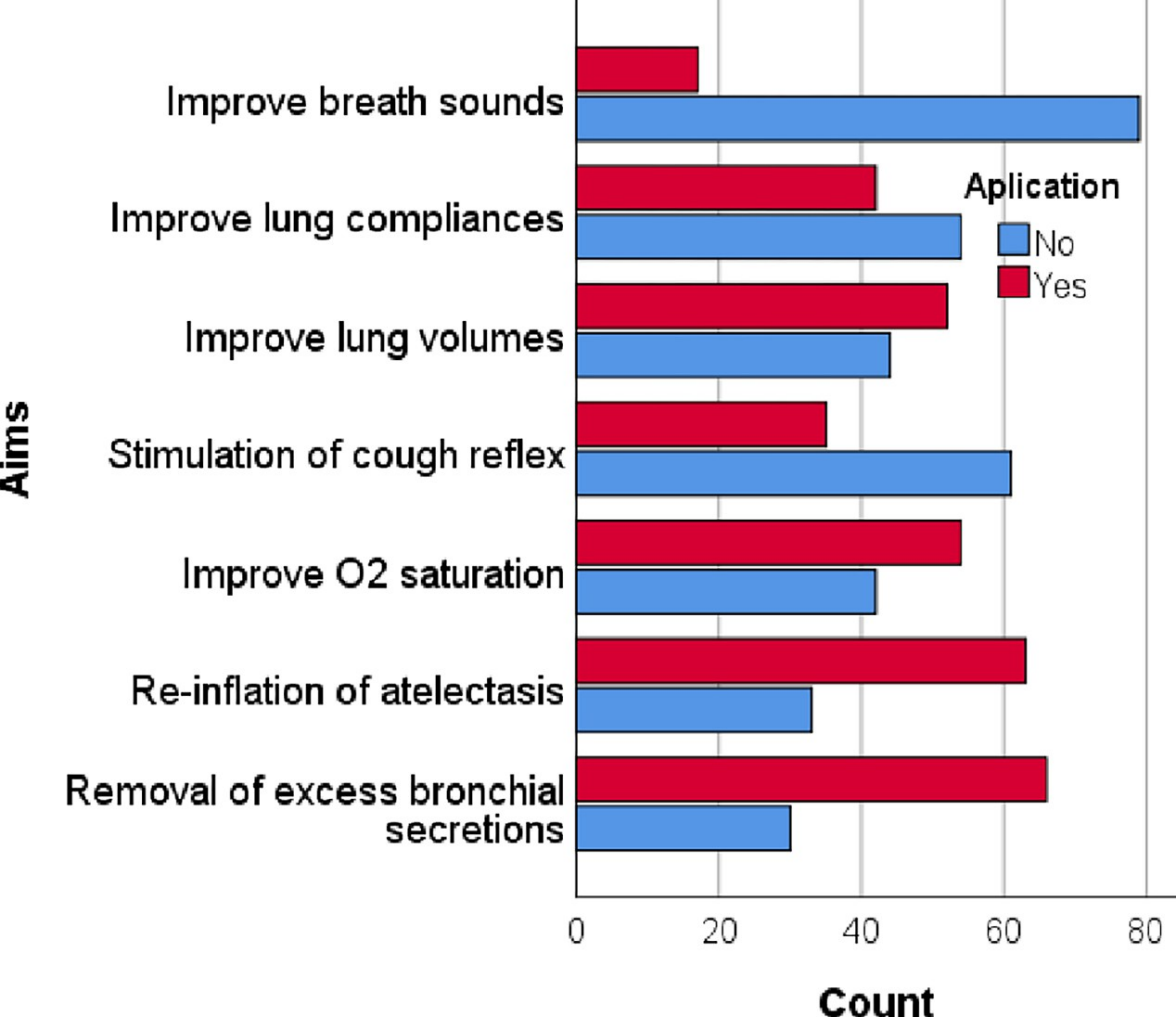
Aims of MHI.

**Fig 3:**
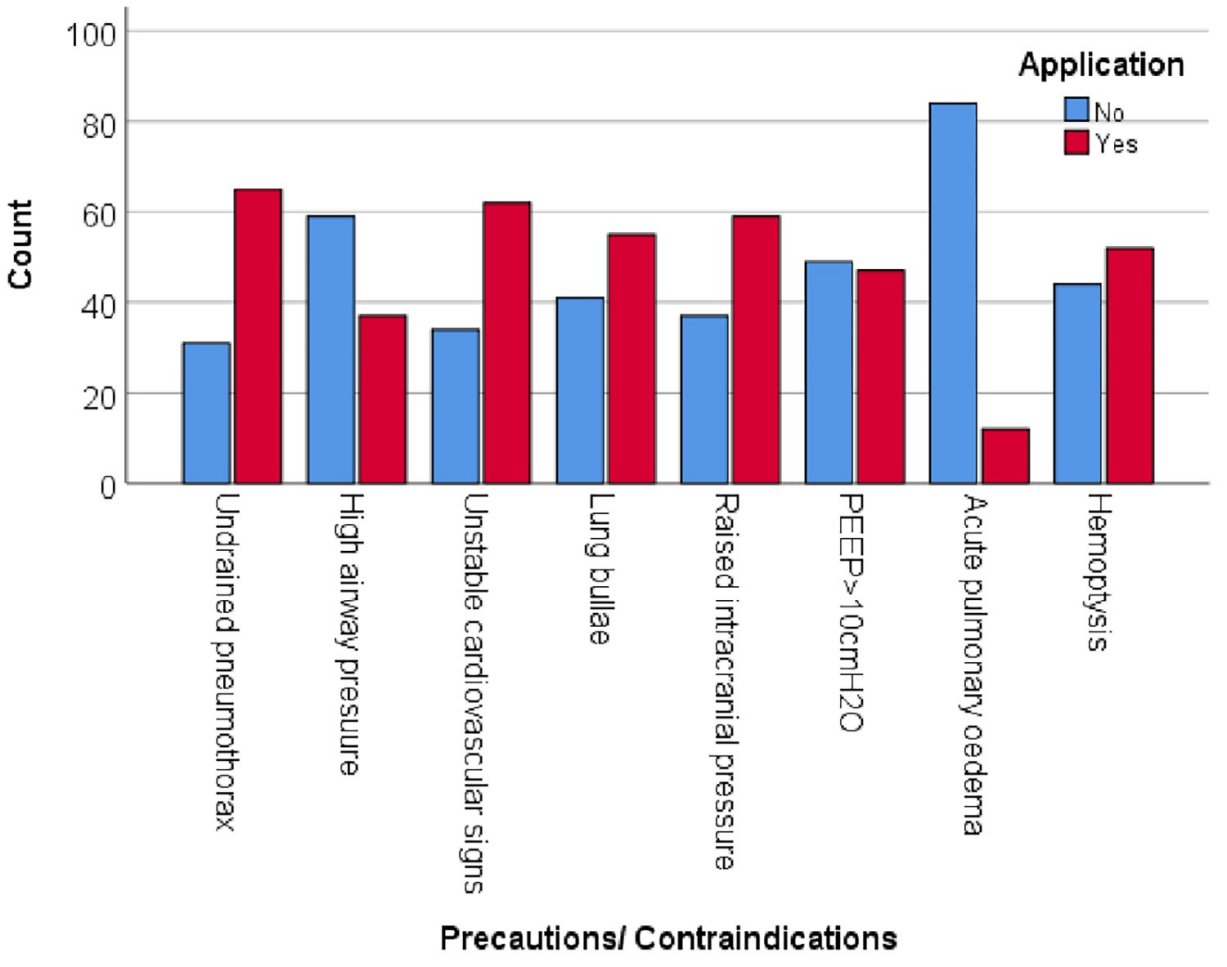
Frequency of precautions and contraindications of MHI considered by the study participants.

From the sample, 90 physiotherapists (93.8%) used hyperinflation in the ICU. Among them, 71 physiotherapists (78.9%) only used MHI, one physiotherapist (1.1%) used VHI and 18 physiotherapists (20%) used MHI and VHI. MHI is used routinely (40.6%) and is also used to treat low oxygen saturation (53.1%), abnormal breath sounds (34.4%), abnormal chest X-ray findings (23.9%), and when the consultant instructed (33.3%) (Fig 1). Participants responded that MHI aimed to improve breath sounds (17.7%), lung compliances (43.6%), lung volumes (54.2%), oxygen saturation (56.3%), stimulation of cough reflex (36.5%), re-inflation of atelectasis (65.6%) and removal of excess bronchial secretions (68.8%) (Fig 2). Undrained Pneumothorax (67.7%), high airway pressure (38.5%), unstable cardiovascular signs (64.6%), lung bullae (57.3%), raised intracranial pressure (61.5%), PEEP >10cmH_2_O (49%), acute pulmonary oedema (12.5%), and hemoptysis (54.2%) were the common contraindications that participants took into account during MHI application (Fig 3).

From the sample, the majority (40.6%) used self-inflation bags, 21.8% used Laerdal 2L, and 17.7% used Mapleson-C. The treatment time for MHI varied from < 5 minutes to 20 minutes and the majority of participants (56.7%) claimed that treatment time depended on the patient’s condition. The number of breaths given in a set during the MHI varied from one to two breaths to >10 breaths and 42.7% did not respond. Only 20.8% of participants responded that the FiO_2_ level during MHI was adjusted between 0.5 litre- one litre while 31.3% did not respond. Among 65 respondents, only 39 (40.6%) used the peak expiratory valve during MHI for PEEP-dependent patients. Among 73 respondents, only 36 (37.1%) used PIP during the application and from 36 respondents, 25 (26%) used a manometer to measure PIP during MHI while others used ventilators. Only 3.1% of the participants reported that PIP was set to <40 cmH_2_O during MHI while 69.8% did not respond. All the participants have used the supine position during treatments and among them, 33 participants (34.4%) used the side-lying position (affected lung up) during the treatments (Table 2).

**Table 2:** Frequencies and percentages of the participants using different types of devices and parameters of MHI (n = 96).

MHI parameters	Frequency (%)
**Device used**	
Self-inflating	39 (40.6)
Laerdal 2L	21(21.8)
Superstring	0(0)
Anaesthetic	17 (17.7)
Mapleson-C	17 (17.7)
**Total duration of the treatment (minutes)**	
<5	7 (10.5)
10	10 (14.9)
15	9 (13.4)
20	3 (4.5)
Depending on the patient	38 (56.7)
N/A	29 (30.2)
**Number of breaths per set**	
1–2	3 (3.1)
3–4	11(11.5)
5–6	6 (6.3)
7–9	9 (9.4)
≥10	21 (21.9)
Depends on the patient	5 (5.2)
N/A	41 (42.7)
**FiO**_**2**_ **(%)**	
0.1–0.49	36 (37.5)
0.5–1	20 (20.8)
Depends on the patient	10 (10.4)
N/A	30 (31.3)
**PIP (cmH**_**2**_ **O)**	
≤40	26 (27.1)
>40	3 (3.1)
N/A	67 (69.8)
**Patient positioning (Yes)**	
Supine	96 (33.3)
Side lying affected lung up	33 (11.5)
Side lying affected lung down	3 (1.0)

**Fig 4:**
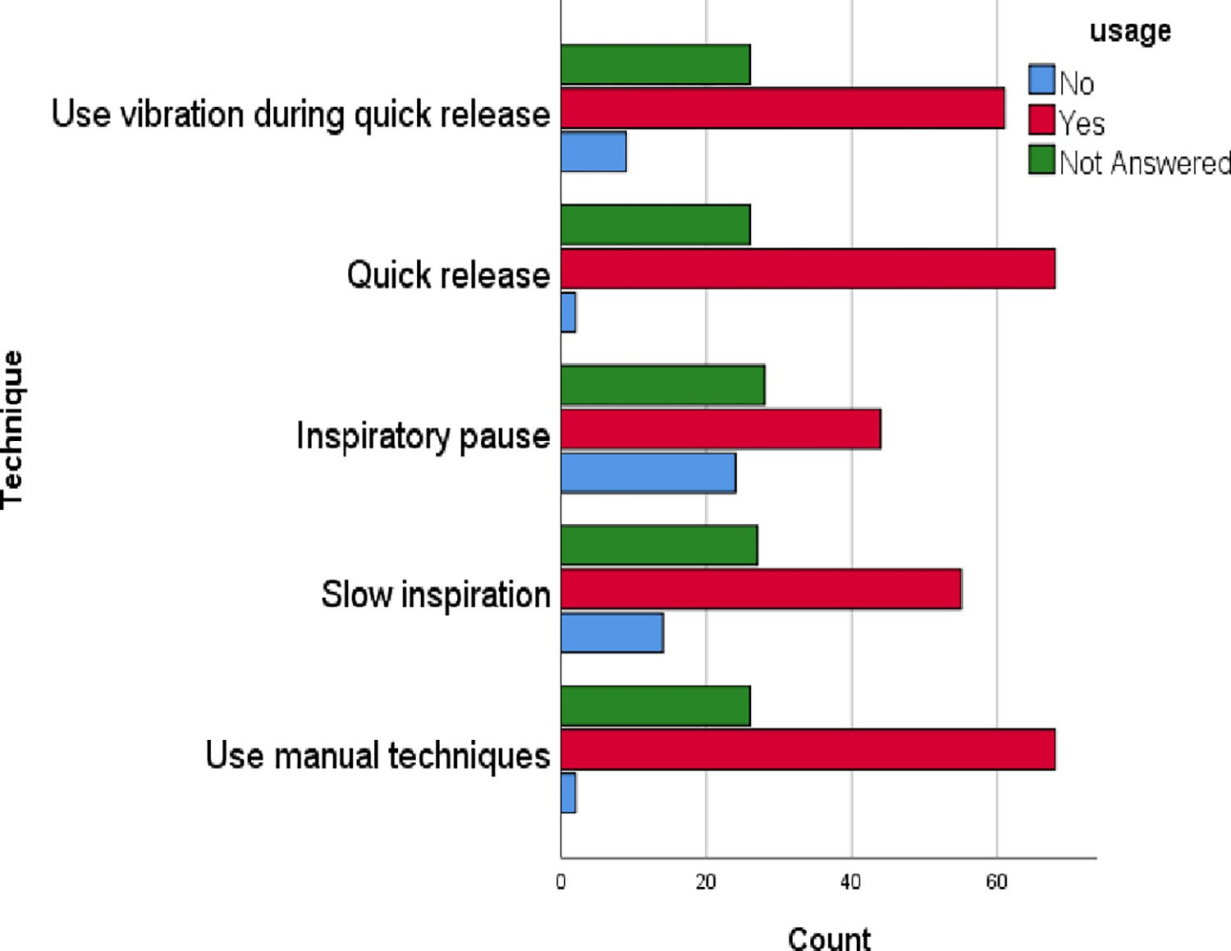
Techniques used in MHI.

When considering the application of technique during MHI, participants reported that they used manual techniques (70.8%), slow inspiration (57.3%), inspiratory pause (45.8%), quick release (70.8%), and used vibration during quick release (63.5%) (Fig 4).

### Practice of VHI

There were 19 (19.8%) participants from the total sample who were practising VHI. Among them, 14 (73.7%) informed that they needed medical approval for VHI at the ICU. They reported that VHI was mostly practised by medical staff (47.4%) while only 21% of physiotherapists practised it (Table 3). Nine (47.4%) physiotherapists practised with a VHI protocol and 7 (36.8%) practised without a VHI protocol. Out of all the participants, 42.1% reported that they rarely practised VHI, while 21.1% reported that they practised it frequently (Table 3). It was found that 10 (52.6%) participants have altered the technique of VHI when treating lung collapse versus sputum retention while 5 (26.3%) participants were using the same technique. Thirteen (68.4%) have practised these techniques as not exceeding the maximum volume while 2 (10.5%) did not consider the maximum volume. The number of sets of deep breathing given during VHI varied from 1 to more than 10 breaths and 57.7% responded as 3–4 sets. The number of breaths for a set also varied from 1 to more than 10 and the majority (31.6%) responded as 3–4 breaths per a set (Table 3).

**Table 3:** Practicing of VHI among the participants (n = 96).

VHI parameters	Frequency (%)
**Staff perform the VHI**	
Senior Physiotherapist	2 (10.5)
Junior Physiotherapist	2 (10.5)
Physiotherapy students	0 (0)
Medical staff	9 (47.4)
Nurses	6 (31.6)
**Frequency of performing VHI**	
Rarely	8 (42.1)
Sometimes	3 (15.8)
Frequently	4 (21.1)
Very Frequently	3 (15.8)
N/A	1(5.3)
**Number. of sets of deep breaths**	
1–2	1 (5.3)
3–4	11 (57.9)
5–10	2 (10.5)
>10	1 (5.3)
N/A	4 (21.1)
**No. of deep breaths per a set**	
1–2	1 (5.3)
3–4	6 (31.6)
5–10	5 (26.3)
>10	4 (21.1)
N/A	3 (15.8)

**Fig 5:**
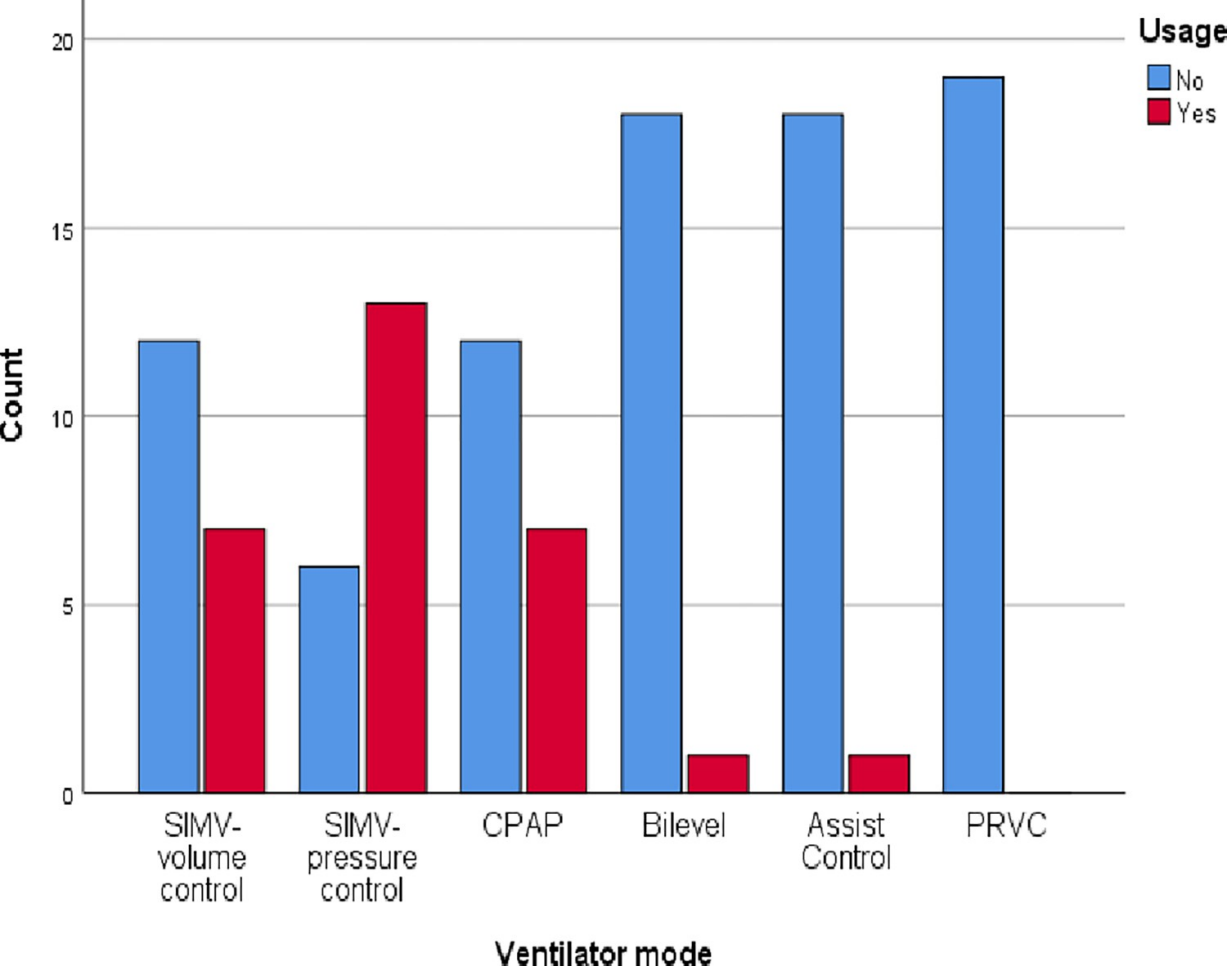
Ventilation mode of VHI.

**Fig 6:**
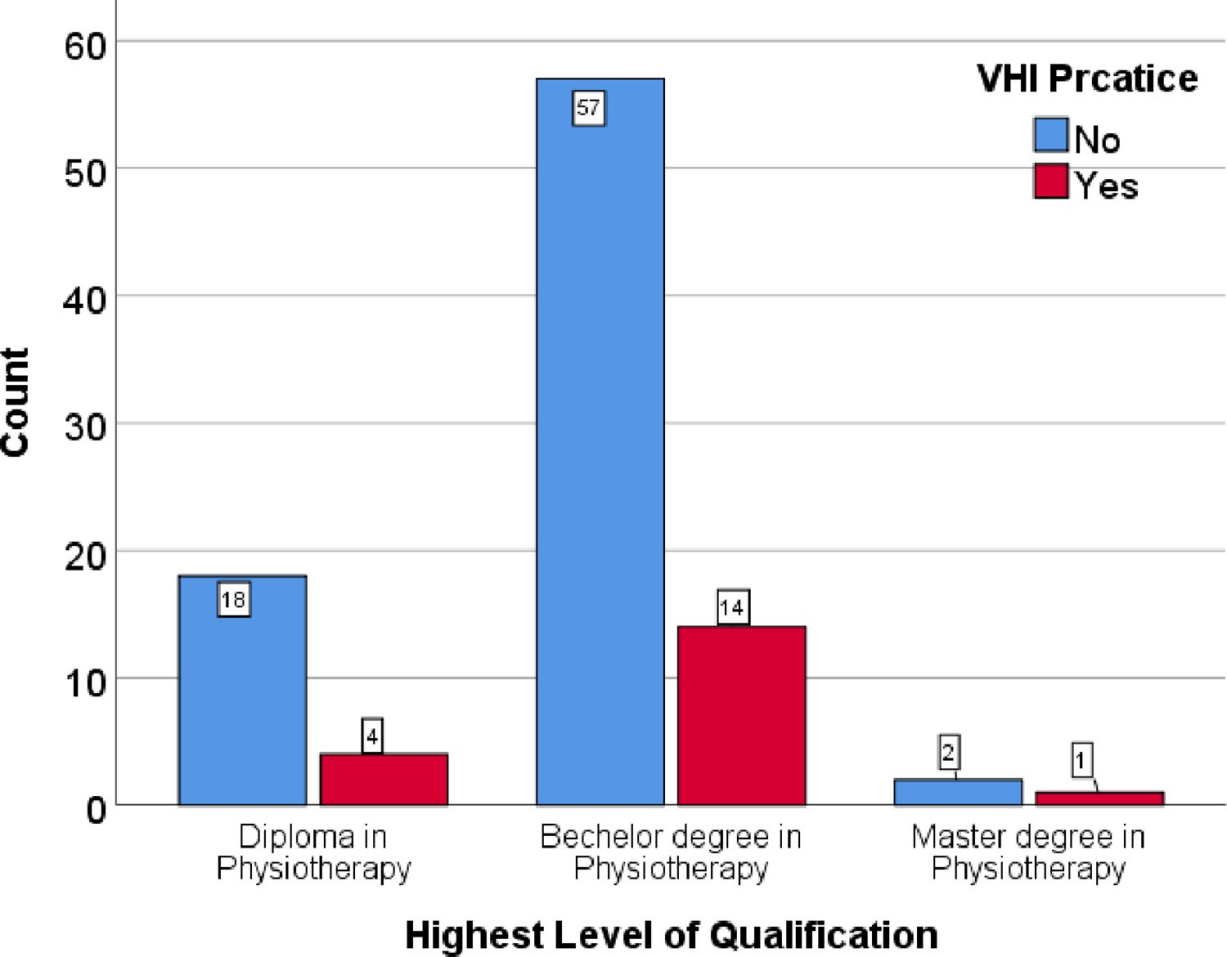
Practice of VHI with education qualification.

**Fig 7:**
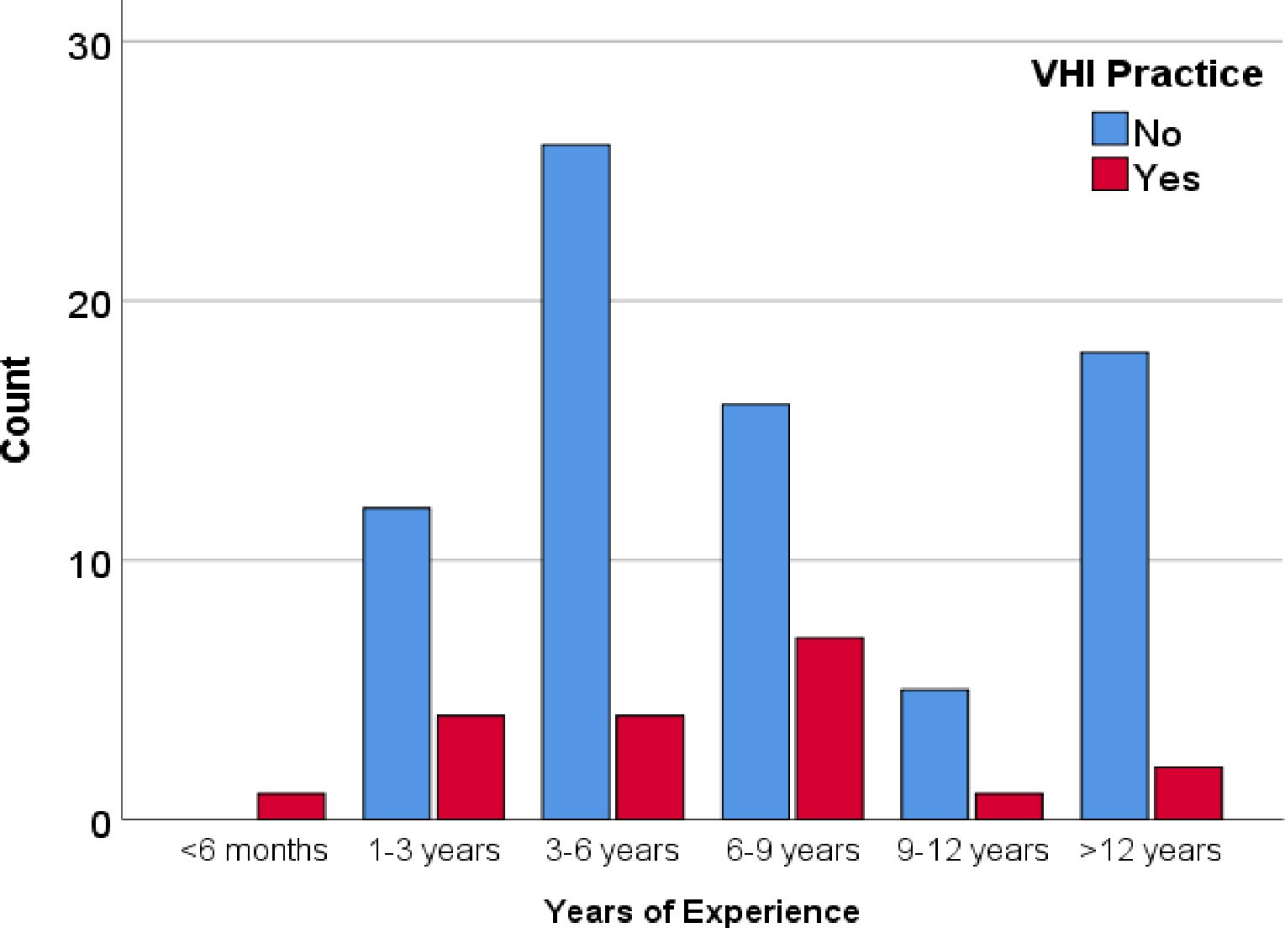
Practice of VHI with years of experience.

During VHI, the respiratory rate should be set and only 5.3% responded as the respiratory rate should be less than 8 while 63.2% did not respond. Only 31.6% of participants reported that the peak pressure that should be set during VHI was <35cmH_2_O while the rest of them did not report any value. Only 10.5% of participants responded that peak volume during VHI was >2L while 73.7% of participants did not respond. No individual has responded that the inspiratory flow rate should be set as 20L/min, while 84.2% did not respond at all about the inspiratory flow rate value. No one responded on the plateau and rise time of VHI. Only 15.8% of participants responded that inspiratory time was set as 5–6 seconds while 84.2% of participants did not report inspiratory time (Table 4). Different ventilatory modes were used during VHI and resonances were as follows; SIMV volume control (36.8%), SIMV pressure control (68.4%), CPAP (36.8%), Bi-level (5.2%), Assist control (5.2%), PRVC (0%) (Fig 5). The survey questioned participants about their source of information on VHI. Of the respondents, 36.8% learned about VHI from their undergraduate program, while 42.1% did not provide a response. Dependency of education level and years of experience practising VHI were measured using chi-squired test. There were no significant impact of eduction level (p = 0.826) or years of experience (p = 0.156) on VHI practice among physiotherapists. Among the physiotherapist who have master degree qualifications 33.3% are practicing VHI. Nevetheless, among the physiotherapist who have diploma qualifications only 18.2% are practicing VHI (Fig 6). Though there is no significant vaiations, those who have working experinces for 6-9years shows higher level of VHI practice compared to others (Fig 7).

**Table 4:** Summary of VHI practice (n = 19).

VHI practices	Frequency (%)
**Set respiratory rate (breaths/min)**	
6–8	1(5.3)
>8	6 (31.6)
N/A	12 (63.2)
**Peak pressure (cm/H**_**2**_ **0)**	
<35	6 (31.6)
35–40	0 (0)
>40	0 (0)
N/A	13 (68.4)
**Peak volume (L)**	
<2	3 (15.8)
>2	2 (10.5)
N/A	14 (73.7)
**Inspiratory flow rate (l/min)**	
<20	3 (15.8)
20	0 (0)
N/A	16(84.2)
**Plateau (sec)**	
2	0 (0)
N/A	19 (100)
**Inspiratory time (sec)**	
<5	0(0)
5–6	3(15.8)
>6	0 (0)
N/A	16 (84.2)
**Ramp/Rise Time (sec)**	
N/A	19 (100)

## Discussion

This study examined the uses and parameters of MHI and VHI among mechanically ventilated patients by intensive care physiotherapists in Sri Lanka. This is the first reported study that examined parameters related to MHI and VHI in Sri Lanka. In this study, the majority of the participants used the hyperinflation technique in ICU. Similar results were observed in several studies in other countries; Australia [16], New Zealand [30] United Kingdom [19] and Brazil [29]

Different devices can be used for MHI. According to our study, self-inflation bags were the most commonly used device, followed by Laerdal 2L and Mapleson-C. However, an Australian study found that the Laerdal bag was most frequently used, followed by the Magill circuit [16]. Meanwhile, a study conducted in the United Kingdom revealed that Water’s bag [19], as well as Laerdal in Hong Kong, were the most frequently used devices for delivering MHI [31]. This variation in usage may be due to availability of devices and education. However, it has been found that the Mapleson C and F circuits resulted in higher PEFR than the Laerdal, which can help facilitate secretion clearance [32].

In the present study, the majority responded that they expected to remove secretion and to reinflate the atelectasis lung followed by improving lung volumes. Similar results were observed in a United Kingdom study where the majority expected to remove secretion followed by reinflating the atelectasis of the lung [19] and in an Australian study the majority of the participants expected to remove secretion and reinflate the atelectasis lung [16]. The benefits expected from MHI are secretion clearance [33], due to expiratory flow bias and reinflation of a collapsed lung occurs as a result of the administration of a larger volume during MHI which opens collapsed alveoli [34].

In the present study majority of the respondents considered undrained pneumothorax as a contraindication and also increased intracranial pressure, unstable cardiovascular signs, lung bullae, PEEP >10cmH_2_O, and acute pulmonary oedema as contraindications which are in consistent with the studies in the United Kingdom [19], Australia [16] and Brazil [29]. To ensure patient safety, it is necessary to consider an undrained pneumothorax or hemothorax as contraindications. Those conditions can increase pleural space air volume, particularly in cases with unidirectional fistulas, leading to heightened lung collapse and a greater risk of hemodynamic instability [29].

In the current study majority performed the prescribed method which facilitated secretion removal and reinflated the lung in intubated patients. These results are consistent with studies conducted in Australia [16] and the UK [19]. MHI application should be performed with a slow squeeze [35], and an inspiratory pause followed by a quick release [14]. Slow squeeze and quick release is a must to create expiratory bias to remove secretion, inspiratory pause to redistribute the air in all the parts of the lung [36] and improve collateral ventilation [19]. This study is incongruent with the study conducted in the Netherlands to assess the practice of MHI among nurses owing to the lack of knowledge or ignorance of the method [37].

In the current study, the majority of participants did not consider PIP during their MHI application and also the majority were not using a manometer to check PIP. It is also observed that accepted PIP varied between 10–100 cmH_2_O among participants. Similar results were observed in studies conducted in Australia [16] and in the UK [19]. The Physiotherapists must utilise a manometer and take PIP into account when administering MHI to prevent barotrauma. It is accepted that PIP should not exceed 40cmH_2_O to avoid barotrauma in the intubated patient [38, 39],

The current study’s respondents indicated treatment times ranging from 5 to 13 minutes, and treatment duration was patient-specific. This is consistent with the broader range of 2 to 45 minutes, with a common choice of 10 minutes reported in an Australian study [16]. In contrast, an experimental study reported notably shorter treatments, averaging two minutes [40] and a study conducted in the Netherlands, reported two to five minutes of treatment time (39. Physiotherapy treatment varies significantly and is influenced by factors such as the patient’s condition and regional practices. This variability often arises due to the absence of standardised guidelines regarding treatment time.

The number of breaths per session in this study showed a wide range, spanning from two to more than ten breaths. Notably, 42.7% of participants did not respond to this question. Similar results were reported in an Australian study where the number of breaths varies from one to fifteen breaths [16]. An experiment study conducted in Spain reported the average number of breaths was 12 [40]. The recommended number of breaths is six breaths per session as a guideline by Maxwell and colleagues [33]. These findings illustrate the diversity and lack of consensus in the field regarding the optimal number of breaths per session.

All the physiotherapist reported to use the supine position for the application of MHI and also side lying on non-affected lung. The positioning of patients in the literature varied from side lying [41–43] to supine [15, 29, 44, 45]. The author’s opinion is that in addition to supine positions due to gravity’s influence, the side-lying position is also useful for improving bronchial drainage during MHI application.

VHI refers to the adjustment of ventilator settings to deliver tidal volumes larger than the baseline, without the drawbacks associated with disconnecting from the ventilator as seen in MHI [22]. In the current study, a percentage of users used VHI in their ICU setting. Among them, the majority of them did not state the parameters they used during the application of VHI. These results are similar to the study conducted in New Zealand and Australia [22]. As VHI is a new approach, it may not be commonly used.

The majority of physiotherapists in the present study responded that they needed medical approval to use VHI. Whereas, Physiotherapists in New Zealand and Australia reported that they do not seek medical approval [22]. The difference in practices across various countries could be the reason for the above finding.

Few Sri Lankan physiotherapists consider setting a PIP or maximum volume delivered during VHI application. Setting PIP is important to prevent barotrauma during hyperinflation [16]. On the contrary the majority of the participants reported that they set a PIP of 40cmH_2_O in a study conducted in New Zealand and Australia [22]. At present, there are no guidelines recommending safe peak airway pressures during VHI [22]. It is important to set PIP less than 40 cmH_2_O to prevent barotrauma.

The number of breaths delivered during application varied from one to more than 10 breaths and the number of sets varied from three to four sets in the present study. In a study conducted jointly in New Zealand and Australia, researchers reported employing five to ten breaths per set and conducting three to four sets [22]. Furthermore, a separate study conducted exclusively in Australia utilised a protocol involving six to eight breaths and four sets [24]. Significant variability exists in the application of VHI concerning the parameters and method involved [3, 46].

The majority of the participants in our study stated that they used SIMV volume control followed by, SIMV pressure control, CPAP. Bi-level, Assist control. This is consistent with a study conducted by Kate and her colleagues [22]. However, literature suggest that volume control mandatory ventilation (VC-CMV) is more effective in secretion clearance than pressure support ventilation (PSV) [26]. However, the most appropriate settings for applying the VHI are not recommended [25].

## Conclusion

In Sri Lankan ICUs, MHI is the predominant hyperinflation technique, yet it is frequently administered with the correct technique but without adhering to the recommended PIP levels, raising concerns regarding patient safety. There exists a notable absence of standardised parameters for VHI. The study indicates a need to educate physiotherapists about current VHI and MHI practice guidelines.

## Strengths and limitations

The study’s sample includes physiotherapists from across the entire country and from various categories of hospitals in Sri Lanka. The lack of response rate for part III (VHI) of the questionnaire is one limitation of the study.

## Supporting information

S1 Checklist Checklist for Reporting Results of Internet E-Surveys (CHERRIES). (DOCX)

S1 File Questionnaire. (PDF)

## References

[1] Tài P, Laurent J, Arthur S, editors. Mechanical ventilation: state of the art. Mayo foundation for medical education and research. Mayo Clin Proc; 2017.

[2] Zisk-Rony RY, Weissman C, Weiss YG. Mechanical ventilation patterns and trends over 20 years in an Israeli hospital system: policy ramifications. Israel journal of health policy research. 2019;8:1–10. 30709421 10.1186/s13584-019-0291-y PMC6357444

[3] Berney S, Denehy L. A comparison of the effects of manual and ventilator hyperinflation on static lung compliance and sputum production in intubated and ventilated intensive care patients. Physiotherapy Research International. 2002;7(2):100–8. Epub 2002/07/12. doi: 10.1002/pri.246 . 12109234

[4] Choi JS-P, Jones AY-M. Effects of manual hyperinflation and suctioning on respiratory mechanics in mechanically ventilated patients with ventilator-associated pneumonia. Australian Journal of Physiotherapy. 2005;51(1):25–30. 15748122 10.1016/s0004-9514(05)70050-7

[5] Hodgson C, Denehy L, Ntoumenopoulos G, Santamaria J, Carroll S. An investigation of the early effects of manual lung hyperinflation in critically ill patients. Anaesthesia and intensive care. 2000;28(3):255–61. doi: 10.1177/0310057X0002800302 10853205

[6] Maa S-H, Hung T-J, Hsu K-H, Hsieh Y-I, Wang K-Y, Wang C-H, et al. Manual hyperinflation improves alveolar recruitment in difficult-to-wean patients. Chest. 2005;128(4):2714–21. doi: 10.1378/chest.128.4.2714 16236947

[7] Paratz J, Lipman J, McAuliffe M. Effect of manual hyperinflation on hemodynamics, gas exchange, and respiratory mechanics in ventilated patients. Journal of Intensive Care Medicine. 2002;17(6):317–24.

[8] Jones AY, Ntoumenopoulos G, Paratz J. Intensive care for the critically ill adult. Physiotherapy for Respiratory and Cardiac Problems. 2008:270–312.

[9] Savian C, Paratz J, Davies A. Comparison of the effectiveness of manual and ventilator hyperinflation at different levels of positive end-expiratory pressure in artificially ventilated and intubated intensive care patients. Heart & Lung. 2006;35(5):334–41. doi: 10.1016/j.hrtlng.2006.02.003 16963365

[10] McCarren B, Chow CM. Manual hyperinflation: a description of the technique. Australian Journal of Physiotherapy. 1996;42(3):203–8. doi: 10.1016/s0004-9514(14)60387-1 11676651

[11] Paulus F, Veelo DP, de Nijs SB, Beenen LF, Bresser P, de Mol BA, et al. Manual hyperinflation partly prevents reductions of functional residual capacity in cardiac surgical patients-a randomized controlled trial. Critical Care. 2011;15(4):1–10. doi: 10.1186/cc10340 21819581 PMC3387630

[12] Tucci MR, Nakamura MA, Carvalho NC, Volpe MS. Manual Hyperinflation: Is It Effective?: Respiratory Care; 2019. p. 870–3. doi: 10.4187/respcare.07152 31243163

[13] Zhao Y, Markides CN, Matar OK, Hewitt GF. Disturbance wave development in two-phase gas–liquid upwards vertical annular flow. International Journal of Multiphase Flow. 2013;55:111–29.

[14] Maxwell LJ, Ellis ER. The effect of circuit type, volume delivered and “rapid release” on flow rates during manual hyperinflation. Australian Journal of Physiotherapy. 2003;49(1):31–8. doi: 10.1016/s0004-9514(14)60186-0 12600252

[15] Rothen HU, Sporre B, Engberg G, Wegenius G, Hedenstierna G. Re-expansion of atelectasis during general anaesthesia: a computed tomography study. BJA: British Journal of Anaesthesia. 1993;71(6):788–95. 8280539 10.1093/bja/71.6.788

[16] Hodgson C, Carroll S, Denehy L. A survey of manual hyperinflation in Australian hospitals. Aust J Physiother. 1999;45(3):185–93. Epub 2001/10/26. doi: 10.1016/s0004-9514(14)60349-4 . 11676766

[17] O’Donnell K. A survey of hyperinflation techniques in ICU based physiotherapists in the UK. Physiotherapy. 2019;105:e178.

[18] Bennett BG, Thomas P, Ntoumenopoulos G. Effect of Inspiratory Time and Lung Compliance on Flow Bias Generated During Manual Hyperinflation: A Bench Study. Respir Care. 2015;60(10):1449–58. Epub 2015/09/10. doi: 10.4187/respcare.04066 . 26350234

[19] King D, Morrell A. A survey on manual hyperinflation as a physiotherapy technique in intensive care units. Physiotherapy. 1992;78(10):747–50.

[20] Ortiz Tde A, Forti G, Volpe MS, Carvalho CR, Amato MB, Tucci MR. Experimental study on the efficiency and safety of the manual hyperinflation maneuver as a secretion clearance technique. J Bras Pneumol. 2013;39(2):205–13. Epub 2013/05/15. doi: 10.1590/S1806-37132013000200012 ; PubMed Central PMCID: PMC4075822. 23670506 PMC4075822

[21] Paulus F, Binnekade JM, Middelhoek P, Vroom MB, SchuItz MJ. Guideline implementation powered by feedback and education improves manual hyperinflation performance. Nurs Crit Care. 2016;21(1):36–43. Epub 2014/05/08. doi: 10.1111/nicc.12068 . 24801958

[22] Kate H, Daniel S, Melissa W. Ventilator hyperinflation: a survey of current physiotherapy practice in Australia and New Zealand. New Zealand Journal of Physiotherapy. 2011;39(3):124.

[23] Main E, Denehy L. Cardiorespiratory Physiotherapy: Adults and Paediatrics: formerly Physiotherapy for Respiratory and Cardiac Problems: Elsevier Health Sciences; 2016.

[24] Dennis D, Jacob W, Budgeon C. Ventilator versus manual hyperinflation in clearing sputum in ventilated intensive care unit patients. Anaesthesia and intensive care. 2012;40(1):142–9. doi: 10.1177/0310057X1204000117 22313075

[25] Ribeiro BS, Lopes AJ, Menezes SLS, Guimarães FS. Selecting the best ventilator hyperinflation technique based on physiologic markers: A randomized controlled crossover study. Heart & Lung. 2019;48(1):39–45. 10.1016/j.hrtlng.2018.09.006. 30336946

[26] Thomas PJ. The effect of mechanical ventilator settings during ventilator hyperinflation techniques: a bench-top analysis. Anaesth Intensive Care. 2015;43(1):81–7. Epub 2015/01/13. doi: 10.1177/0310057X1504300112 . 25579293

[27] Sigera PC, Tunpattu TM, Jayashantha TP, De Silva AP, Athapattu PL, Dondorp A, et al. National Profile of Physical Therapists in Critical Care Units of Sri Lanka: Lower Middle-Income Country. Phys Ther. 2016;96(7):933–9. Epub 2016/02/20. doi: 10.2522/ptj.20150363 . 26893503

[28] Eysenbach G. Improving the quality of Web surveys: the Checklist for Reporting Results of Internet E-Surveys (CHERRIES). J Med Internet Res. 2004;6(3):e34. Epub 2004/10/09. doi: 10.2196/jmir.6.3.e34 ; PubMed Central PMCID: PMC1550605. 15471760 PMC1550605

[29] Cruz RVS, Andrade FdSdSDd, Menezes PDGd, Gonçalves BO Almeida RdS, Santos AR. Manual hyperinflation and the role of physical therapy in intensive care and emergency units. Fisioterapia em Movimento. 2017;30:241–8.

[30] Reeve J, Munn L, Quinn L, Eagle E, Jenson L. The use of manual and ventilator hyperinflation by physiotherapists in New Zealand intensive care units. 2008.

[31] Luadsri T, Boonpitak J, Pongdech-Udom K, Sukpom P, Chidnok W. Immediate effects of manual hyperinflation on cardiorespiratory function and sputum clearance in mechanically ventilated pediatric patients: A randomized crossover trial. Hong Kong Physiotherapy Journal. 2022;42(01):15–22. doi: 10.1142/S1013702522500020 35782699 PMC9244603

[32] Hodgson C, Ntoumenopoulos G, Dawson H, Paratz J. The Mapleson C circuit clears more secretions than the Laerdal circuit during manual hyperinflation in mechanically-ventilated patients: a randomised cross-over trial. Aust J Physiother. 2007;53(1):33–8. Epub 2007/03/01. doi: 10.1016/s0004-9514(07)70059-4 . 17326736

[33] Maxwell LJ, Ellis ER. Pattern of ventilation during manual hyperinflation performed by physiotherapists. Anaesthesia. 2007;62(1):27–33. Epub 2006/12/13. doi: 10.1111/j.1365-2044.2006.04898.x . 17156224

[34] Tweed WA, Phua WT, Chong KY, Lim E, Lee TL. Large tidal volume ventilation improves pulmonary gas exchange during lower abdominal surgery in Trendelenburg’s position. Can J Anaesth. 1991;38(8):989–95. Epub 1991/11/01. doi: 10.1007/BF03008617 . 1752022

[35] Clement A. Chest physiotherapy by the bag squezing method. Physiotherapy. 1968;54:355–9. 5708074

[36] Paulus F, Binnekade JM, Vroom MB, Schultz MJ. Benefits and risks of manual hyperinflation in intubated and mechanically ventilated intensive care unit patients: a systematic review. Crit Care. 2012;16(4):R145. Epub 2012/08/07. doi: 10.1186/cc11457 PubMed Central PMCID: PMC3580733. 22863373 PMC3580733

[37] Paulus F, Binnekade JM, Middelhoek P, Schultz MJ, Vroom MB. Manual hyperinflation of intubated and mechanically ventilated patients in Dutch intensive care units—a survey into current practice and knowledge. Intensive Crit Care Nurs. 2009;25(4):199–207. Epub 2009/05/30. doi: 10.1016/j.iccn.2009.04.003 . 19477647

[38] Marcy TW. Barotrauma: detection, recognition, and management. Chest. 1993;104(2):578–84. doi: 10.1378/chest.104.2.578 8339650

[39] Haake R, Schlichttg R, Ulstad DR, Henschen RR. Barotrauma: pathophysiology, risk factors, and prevention. Chest. 1987;91(4):608–13. 3549176 10.1378/chest.91.4.608

[40] Bassi GL, Martí JD, Comaru T, Aguilera-Xiol E, Rigol M, Ntoumenopoulos G, et al. Short-term appraisal of the effects and safety of manual versus ventilator hyperinflation in an animal model of severe pneumonia. Respiratory Care. 2019;64(7):760–70. doi: 10.4187/respcare.06487 31088989

[41] Scholten DJ, Novak R, Snyder JV. Directed manual recruitment of collapsed lung in intubated and nonintubated patients. Am Surg. 1985;51(6):330–5. Epub 1985/06/01. . 3994176

[42] Novak RA, Shumaker L, Snyder JV, Pinsky MR. Do periodic hyperinflations improve gas exchange in patients with hypoxemic respiratory failure? Crit Care Med. 1987;15(12):1081–5. Epub 1987/12/01. doi: 10.1097/00003246-198712000-00001 . 3677760

[43] Stiller K, Geake T, Taylor J, Grant R, Hall B. Acute lobar atelectasis. A comparison of two chest physiotherapy regimens. Chest. 1990;98(6):1336–40. Epub 1990/12/01. doi: 10.1378/chest.98.6.1336 . 2245671

[44] Singer M, Vermaat J, Hall G, Latter G, Patel M. Hemodynamic effects of manual hyperinflation in critically ill mechanically ventilated patients. Chest. 1994;106(4):1182–7. Epub 1994/10/01. doi: 10.1378/chest.106.4.1182 . 7924493

[45] Tweed WA, Phua WT, Chong KY, Lim E, Lee TL. Tidal volume, lung hyperinflation and arterial oxygenation during general anaesthesia. Anaesth Intensive Care. 1993;21(6):806–10. Epub 1993/12/01. doi: 10.1177/0310057X9302100610 . 8122738

[46] Ahmed F, Shafeeq AM, Moiz JA, Geelani MA. Comparison of effects of manual versus ventilator hyperinflation on respiratory compliance and arterial blood gases in patients undergoing mitral valve replacement. Heart Lung. 2010;39(5):437–43. Epub 2010/06/22. doi: 10.1016/j.hrtlng.2009.10.006 . 20561856

