# Supplementary material for: Manual and ventilator hyperinflation parameters used by intensive care physiotherapists in Sri Lanka: An online survey
Source: PLoS One. 2024 May 20;19(5):e0297880. doi: 10.1371/journal.pone.0297880 (PMC11104683; doi:10.1371/journal.pone.0297880)
Supplement: S1 File — (PDF) [file pone.0297880.s002.pdf]

Questionnaire

A survey to assess the MHI and VHI technique in Sri Lanka

Thank you for your participation. The aim of this survey is to collect information related to the use of Manual and Ventilator hyperinflation among physiotherapists in Sri Lankan intensive care units.

I have read the above information and I consent voluntarily to participate as a participant in this research. I know that the data I am providing will be protected and my privacy will be maintained. I know that no harm will happen by participating in this survey. I give my consent to participate in this study

Yes

No (Submit the form)

Demographic data (Part A)

1. Type of hospital where you are employed

   Mark only one

   National Hospital

   General Hospital

   Provincial General Hospital

   Hospital Base,

   Maternal hospitals Government

   Private Hospital

2. How many years have you been working as a physiotherapist

   Mark only one

   < 6 months

   0.5 -1 year

   1-3 Years

   3-6 years

   6-9 years

   9-12 years

   >12 years

3. How many years of experience that you have been working in Intensive Care?

Mark only one

<6 months

0.5-1 Year

1-3 Years

3-6 years

6-9 years

9-12 years

>12 years

4. Your Highest qualification

Mark only one oval.

Diploma in physiotherapy

Bachelor degree in physiotherapy

Master degree in physiotherapy

PhD or Doctor of physiotherapy

5. Are you using hyperinflation as a physiotherapy treatment technique?

Mark only one oval.

Yes

If yes move to question 6

No (Submit the form)

Hyperinflation technique

6. Method used for hyperinflation

   Mark only one oval.

   Manual Hyperinflation
   Move to question 7
   Mechanical /Ventilator Hyperinflation
   Move to question 21

Manual Hyperinflation technique (Part-B)

7. Person responsible for performing hyperinflation technique in your ICU setting

   Mark only one oval.
   Physiotherapist
   Nursing officer
   Medical officer
   Other:

8. Indication or selection criteria to uses hyperinflation

   Tick all that apply.

   Routine procedure
   Reduce the level of oxygen saturation
   Abnormal breath sound (crackle or Ronchi)
   abnormal X-ray finding
   Instruction from consultant doctor or Medical officer
   Other:

9. Aim/s of the hyperinflation technique is

Tick all that apply.

Removal of excess bronchial secretions

Re-inflation of atelectasis / collapsed lung

Improve arterial blood gas tensions and oxygen saturations

Stimulation of a cough reflex

Improve lung volumes

Improve lung compliance/peak airway pressures

Improve breath sounds on auscultation

10. Type of device used for Manual Hyper Inflation

Tick all that apply.

Self-inflating

Laerdal 2L

Supersyring~

Anesthetic

Mapleson-C

Other:

11. Fraction of inspired Oxygen (Fi02) used for MHI

…………

12. Numbers of breath per set used during the application of MHI

……………..

13. Total duration of the treatment

Mark only one oval.

<5

10 minutes

15 minutes

20 minute

>20 minutes

Depend upon the patient

14. Position of the patient during the MHI procedure

Mark only one oval.

Supine

Side lying affected lung up

side lying affected lung down

15. Precautions and contraindications for MHI

Mark only one oval.

Undrained pneumothorax

High airways pressure

Unstable cardiovascular signs

Lung bullae

Raised Intracranial pressure

PEEP> 10cm Hp

Acute pulmonary oedema

Hemoptysis

Other:

16. Technique used in MHI

	Yes	No
Use Manual techniques (Chest percussion/vibration/shaking) prior to MHI		
Slow inspiration		
Inspiratory Pause		
Quick release		
Use Vibration during Quick release phase		

Mark only one oval per row.

17. Do you use peak expiratory valve during MHI for PEEP dependent patient

Mark only one oval.

Yes

No

18. Do you set/consider Maximum Inspiratory pressure /Peak Inspiratory pressure during the application

Mark only one oval.

Yes

Move to question 19

No

Peak Inspiratory pressure

19. How do you measure Peak inspiratory pressure during MHI

Mark only one oval.

Manometer

Other:

20. What is Maximum Peak Inspiratory pressure used in your ICU

……………………………….

Ventilator Hyperinflation technique (Part C)

21. Do you need medical approval to perform Ventilator Hyperinflation (VHI)?

    Mark only one oval.

    Yes

    No

22. Please indicate which of the following staff perform VHI in your ICU

    Mark only one oval.

    Senior Physiotherapist

    Junior Physiotherapist

    Physiotherapy student

    Medical staff

    Nurse

23. Do you have a VHI protocol

    Mark only one oval.

    Yes

    No

24. How would you describe the frequency that you perform VHI in ICU

Mark only one oval.

Rarely (less than once a month)
Sometimes (less than once a week)
Frequently (more than once a week)
Very Frequently (daily)

25. What do you consider to be the main indications for performing VHI?

…………………………………….

26. What are your main reasons for choosing to perform VHI instead of manual hyperinflation?

……………………………………..

27. Please indicate in which of the following modes of ventilation you would commonly perform VHI

Tick all that apply.

SIMV – volume control
SIMV – pressure control
CPAP/Pressure support mode
Bilevel
Assist Control
PRVC
Other:

28. Please outline below, how you would most commonly perform VHI in your ICU. Please include as much detail as is appropriate for your chosen technique (please note you do not have to fill out information for all the following headings, only those appropriate for your chosen technique):

Mode of ventilation

……………………….

Set Respiratory Rate

…………………………….

Peak pressure

…………………………….

Peak volume

…………………………………….

Inspiratory flow rate

………………………………….

Plateau (sec)

…………………………….

Inspiratory Time (sec)

……………………………..

Ramp/Rise Time

…………………….

29. Do you alter your technique of VHI when treating lung collapse versus sputum retention?

Mark only one oval.

Yes

No

Other:

30. Do you have a maximum pressure or maximum volume that you do not exceed during VHI?

Mark only one oval.

Yes

No

Other:

31. During an average treatment, how many deep breaths do you provide in one set?

Mark only one oval.

1-2

3-4

5-10

>10

32. During an average treatment, how many sets of deep breathes do you provide?

Mark only one oval.

1-2

3-4

5-10

>10

33. How did you learn the practical aspects of performing the technique of VHI?

Mark only one oval.

Taught how to perform VHI as an undergraduate

Taught how to perform VHI at a professional development course

Taught at the bedside by a senior physiotherapist

Taught at the bedside by a medical colleague

Self-taught through reading of scientific literature

Other:

Thank you for your participation
